# Prevalence of Ten Gene Variants Involved in Muscular Phenotypes in a Mexican Mestizo Population

**DOI:** 10.3390/muscles2040030

**Published:** 2023-12-08

**Authors:** Luz Berenice López-Hernández, Guillermina Avila-Ramírez, Ariadna Del Villar-Morales, Mónica Alejandra Anaya-Segura, Luis Angel Montes-Almanza, Froylan Arturo García-Martínez, Antonio Miranda-Duarte, Carlos Antonio Sosa-Flores, Martha Eunice Rodríguez-Arellano, Ileana Chavez-Maisterra, Alexandra Berenice Luna-Angulo, Miriam Pavelth Casillas-Ávila, Benjamín Gómez-Díaz

**Affiliations:** 1 Departamento Académico de Ciencias Clínicas, Universidad Autónoma de Guadalajara, Av Patria 1201, Zapopan 45129, Mexico; ileana.chavez@edu.uag.mx; 2 Asociación de Distrofia Muscular de Occidente A.C., Guadalajara 44380, Mexico; lab@biodna.mx; 3 Facultad de Medicina, Universidad Nacional Autónoma de México, Ciudad de México 04510, Mexico; gavilaramirez@facmed.unam.mx; 4 Instituto Nacional de Rehabilitación Luis Guillermo Ibarra Ibarra, Ciudad de México 14389, Mexico; ariadna@clinicadeldeporte.mx (A.D.V.-M.); amiranda@inr.gob.mx (A.M.-D.); inu-chrono@hotmail.com (C.A.S.-F.); abluna@inr.gob.mx (A.B.L.-A.); 5 BioDNA Laboratory Services Ltd., Zapopan 45010, Mexico; 6 Departamento de Genética, Instituto de Oftalmología ‘Conde de Valenciana’, Ciudad de México 41700, Mexico; luis.montes@condeinvestigacion.org (L.A.M.-A.); froylan.garcia@condeinvestigacion.org (F.A.G.-M.); 7 Departamento de Medicina Genómica, Hospital Regional “Lic. Adolfo López Mateos”, Instituto de Seguridad y Servicios Sociales de los Trabajadores del Estado, Ciudad de México 01030, Mexico; issstecdmx@humanvariomeprojectmexico.org.mx; 8 Hospital Civil “Dr. Antonio González Guevara”, Tepic 63169, Mexico; insalud@humanvariomeprojectmexico.org.mx

**Keywords:** Hardy–Weinberg equilibrium, Mexican Mestizo population, sample size, exercise adaptation, complex metabolic diseases

## Abstract

Several reports have provided evidence that there are genetic variants of genes such as *MSTN*, *BDRKB2*, *ACTN3* and *ADRB2* that are involved in a better response to adaptation during resistance or strength training, while other genes such as *GRB14*, *AGT* and *END1* are reported to be associated with the risk of suffering from some diseases such as diabetes, hypertension or obesity. A cross-sectional study from a Mexican Mestizo population was performed to estimate the frequency of 10 gene variants in 8 genes involved in athletic performance or chronic degenerative diseases, *MSTN* (rs1805085, rs1805086), *BDKRB2* (rs1799722), *FST* (rs1423560), *ACTN3* (rs1815739), *ADRB2* (rs1042713, rs1042714), *GRB14* (rs8192673), *AGT* (rs699) and *EDN1* (rs5370), and to compare frequencies from 26 populations reported in the Database of 1000 Genomes project. Genotype frequencies fitted the Hardy–Weinberg equilibrium, except for *MST* rs1805086 and *FST* rs1423560, and our study revealed significant differences in the distribution of frequencies of some of these gene variants among populations reported in the 1000 Genomes Project. Our findings provide insights regarding the genetic background of our population, and future case–control studies can be carried out with more accurate sample sizes for genetic association studies. Our results may be also useful in recognizing the roles and mechanisms contributing to athletic performance and/or chronic degenerative diseases in Mexicans.

## 1. Introduction

Genetic variations significantly impact human muscle structure and function. These differences can lead to distinct anatomical, metabolic, functional, and behavioral characteristics in individuals. Some individuals may possess traits that make them particularly adept at certain physical activities or sports. In contrast, others might be more prone to chronic degenerative diseases like hypertension, diabetes and obesity (Table 1). Understanding these genetic influences can provide valuable insights into human health and performance [1,2]. The diversity observed in the characteristics of skeletal muscle among individuals can be traced back to a combination of genetic factors, environmental influences, or an interplay of both. The role of genetics in determining muscle strength has been hinted at in studies involving twins, which have shown heritability estimates for muscle strength traits to be between 30% and 85%. These estimates, however, can vary based on the specific conditions under which muscle strength is measured. These conditions could include factors such as the limb involved, the angle of contraction, the speed of contraction, and the type of contraction. Despite these findings, the most compelling evidence for a genetic influence on muscle function has been provided by genetic association studies. These studies have shown that variations within an individual’s genome can significantly impact muscle function. These variations can manifest in several ways. They could be as subtle as changes in a single base within the DNA sequence or could involve small deletions and insertions in the sequence. More significant structural variations are also possible, such as alterations in the copy number (changes affecting 50 or more base pairs) [3]. Single nucleotide polymorphisms (SNPs) are the most prevalent and the most thoroughly studied. A single nucleotide variant has the capacity to introduce a stop codon, create a different protein, or even disrupt the secondary structure of the DNA molecule. These intricacies in DNA and the dynamic nature of the genome have been thoroughly explored in previous studies [4,5,6,7].

A recent review of the effects of genetic variations on endurance performance, muscle strength, and susceptibility to injury in competitive sports found that certain genetic variants are overrepresented in elite athletes. One such variant is the rs1464430 (c.275124 A > C) variant of the Insulin Like Growth Factor 1 Receptor gene (IGF1R), which is a critical regulator of muscle growth and repair. The IGF-1 signaling pathway is activated when IGF-1 binds to the IGF1R on the surface of muscle cells. This activates a cascade of signaling events that leads to increased protein synthesis and muscle growth. The rs1464430 (c.275124 A > C) variant of the IGF1R gene is associated with increased IGF1R expression. This means that people with this variant have more IGF1Rs on their muscle cells, which makes their cells more sensitive to IGF-1. This increased sensitivity to IGF-1 can lead to increased muscle growth and endurance. Studies have shown that the rs1464430 (c.275124 A > C) variant is more common in elite endurance athletes than in the general population. This suggests that this variant may play a role in determining who is well-suited for endurance sports. However, it is important to note that genetic variations are just one factor that influences athletic performance. Other factors, such as training, nutrition, and recovery, also play an important role [8].

In addition, the Alpha-actinin-3 gene (ACTN3), which is one of the genes that are linked to improved endurance performance, may also be connected to a higher risk of injuries. For instance, the TT genotype (XX variant) of the rs1815739 polymorphism (c.1729C > T) on the ACTN3 gene has been proven to enhance endurance exercise performance. However, other studies have indicated that this specific genotype is also associated with a higher likelihood of musculoskeletal tissue injury and exercise-induced muscular damage [9].

Hence, phenotypic variance is assumed to be influenced by genetic variations, which are known to differ across various ethnicities and population origins. Consequently, acquiring an understanding of the prevalence of these genetic variants within the adaptive and evolutionary context of the study population is critically important for conducting genetic association studies with sufficient statistical power and for obtaining reliable data that truly represent a population [10]. Phenomena such as bottlenecks, admixture, migrations, and adaptation to environmental conditions may influence gene variant frequencies in specific populations. Interestingly, a recent study highlighted ethnic differences in fatty-acid-derived desaturation indices, wherein insulin-resistant Black women exhibited a fatty acid pattern characteristic of higher insulin sensitivity in European populations [11]. Therefore, the polygenic profile associated with the muscle phenotypes of certain population groups emerges as an approach to investigate endurance in elite athletes and performance in professional sports by comparing allele and genotypic frequencies with the non-athletic population [12].

In general, increasing evidence suggests that particular variants in some genes such as rs1805085 and rs1805086 in the Myostatin gene (MSTN), rs1799722 in the Bradykinin Receptor B2 gene (BDKRB2), rs1423560 in the Follistatin gene (FST), rs1042713 and rs1042714 in the Adrenoceptor Beta 2 gene (ADRB2) and rs1815739 in the ACTN3 gene are involved in differential muscle adaptation to strength and endurance training among individuals. Variants such as rs8192673 in the Growth Factor Receptor Bound Protein 14 gene (GRB14), rs699 in the Angiotensinogen gene (AGT) and rs5370 in the Endothelin 1 gene (EDN1), on the other hand, are associated with chronic degenerative diseases like type 2 diabetes (T2D), essential hypertension (HT) and obesity (Table 2). The prevalence of genetic variants affecting muscular and metabolic phenotypes is largely unknown in Mexican Mestizos as a population group.

The Mexican Mestizo population is the largest group of people in present-day Mexico. This group includes individuals whose parents were born in Mexico and who do not belong to a specific indigenous population. Thus, certain factors such as small population size, geographical isolation, and consanguineous marriages within specific indigenous populations may have an impact on biomedical traits [27]. In this study, we conducted a thorough analysis of a series of genetic variants known to influence muscular performance in Mexican Mestizos. This is the first study to examine these genetic variants in this particular population group.

## 2. Results

This cross-sectional study included a total of 282 Mexican Mestizo individuals, comprising 195 males and 87 females (Table 1). The primary objective of this study was to meticulously analyze the frequency of ten distinct gene variants. These variants are found within eight genes that have been previously associated with athletic performance or chronic degenerative diseases. Additionally, the frequencies were compared to 26 other populations around the world using information sourced from the 1000 Genomes Project [28]. These 26 populations were chosen because of the availability of data to compare the 10 polymorphisms included in this study. Herein, we examined the following gene variants: rs1805085 and rs1805086 of *MSTN*, rs1799722 of *BDKRB2*, rs1423560 of *FST*, rs1815739 of *ACTN3*, rs1042713 and rs1042714 of *ADRB2*, rs8192673 of *GRB14*, rs699 of *AGT* and rs5370 of *EDN1*.

No statistically significant sex differences were detected in any of the variables studied, except for alcoholism and smoking (Table 1).

In our study, we sought to understand the dynamics of allele and genotype frequencies within our target population. To achieve this, we employed the Hardy–Weinberg Equilibrium (HWE) as a theoretical framework. The HWE postulates that both allele and genotype frequencies in a population would remain constant from generation to generation in the absence of other evolutionary influences.

Our first step was to estimate the number of homozygous and heterozygous carriers within the population. This was based on the observed allele frequencies. We then conducted a Chi-square test that was used to determine whether there was a significant difference between the observed genotype frequencies in our population and what would be expected under the HWE.

The results indicated that allele and genotype frequencies were generally consistent with HWE (Table 3) for most of the studied variants. However, exceptions were observed in the rs1805086 of *MSTN* (*p* = 0.024) and rs1423560 of *FST* (*p* < 0.01). These two SNPs exhibited significant differences in comparison to some American populations, as well as to all African populations. For *FST* rs1423560 and *EDN1* rs5370, the most substantial differences were noted in Asian and European populations, while *BDKRB2* rs19799722 and *ACTN3* rs1925739 displayed differences across African, American, Asian and European populations. Notably, *ADRB2* rs1042713 and rs1042714 exhibited more prominent differences with European populations, *GRB14* rs8192673 displayed variations in relation to all Asian populations and *AGT* rs699 demonstrated differences across almost all African, Asian, American and European populations (Supplementary Material).

## 3. Discussion

The study of genetic variations linked to human muscle structure and function may have diverse implications, particularly in fields like sports medicine. Genetic case–control studies are a powerful tool for identifying genetic variants associated with complex diseases. The basic idea is to compare the allele frequencies of genetic markers between cases and controls. This holds true for both pathological conditions (e.g., diabetes, hypertension, and sarcopenia) and positive traits (e.g., muscle resistance in high-performance athletes) [10]. If a marker is associated with the disease or the feature of interest (characteristics of high-performance athletes), its allele frequency will be higher in cases than in controls. However, it is important to be aware of potential biases that can affect the results of genetic case–control studies. One of these biases is population stratification. If the cases and controls come from different populations with different allele frequencies, this can lead to false-positive associations [29]. Thus, in order to avoid spurious associations, researchers should consider population origins, substructure, admixture, and the statistical power obtained. Statistical power, defined as the probability to reject a null hypothesis (H0) whilst the alternative hypothesis (HA) is correct, is affected by sample size, the prevalence of the disease, the frequency of the genetic variant and the assumed inheritance model, among other factors [30].Therefore, examining genotype and allele frequencies within a specific population may be useful for two main purposes: (1) gaining insights into the genetic background of that specific population, (in this case, Mexican Mestizos, representing most present-day Mexicans) [29] and (2) enhancing the accuracy of case–control association studies. The statistical power of a genetic association study is influenced by the disease (trait) prevalence, allele frequency, risk conferred by the genotype and the sample size and the genetic model selected. These factors will generally increase the statistical power of the study. However, the relationship between these factors is not linear [31] (Supplementary Material).

In addition, another potential bias is Hardy–Weinberg disequilibrium (HWD). HWD occurs when the allele frequencies of a marker do not follow the expected proportions [32]. This can happen for a number of reasons, such as selection, inbreeding, or genotyping errors. Our study identified a notable departure from the HWE for the rs1423560 variant. This specific variant is situated in the 5′ untranslated region (5′ UTR) of the FST gene, in proximity to several transcription binding sites. Given its conservation and location, there is a strong indication that this variant is involved in gene transcription. Indeed, this particular variant was found to influence baseline muscle size and strength among African Americans [16]. It is noteworthy that the rs1423560 variant was described to fit the HWE in two populations of Caucasians from Perth, Western Australia, and from the US [16,33]. But when data from the HapMap project were consulted, the ASW (n = 98) group derived from African ancestry in southwestern USA was not in HWE [34]. Therefore, caution should be taken when performing genetic association studies.

Interestingly, a study by Curiel-Cervantes et al. showed that the *MSTN* SNP rs1805086 was linked to higher levels of body mass index (BMI) and waist/height ratio (WHtR) in heterozygous subjects, who showed a greater total and central obesity compared to the homozygous subjects for ancestral allele AA (OR BMI > 30 kg/m). Remarkably, the genotype frequency of AG was 5.4%, and the homozygous risk allele GG was absent (*n* = 1024), and fitted the HWE, which contrasts with the data found by the present study (Table 3) [35]. The discordance found between the study of Curiel-Cervantes et al. [35] and our study is unlikely to be the result of genotyping errors because real-time PCR is accurate for SNP genotyping; nonetheless, it could be a result of the difference in sample size between both studies.

Therefore, the wealth of data presented here enables more precise and larger studies with selected phenotypes regarding exercise adaptation and complex metabolic diseases in Mexican Mestizos. The allele frequencies and Hardy–Weinberg equilibrium (HWE) results presented here can be used by other researchers to design and conduct case–control genetic association studies in Mexican Mestizo populations for traits such as sarcopenia, obesity, muscle size, and strength response to resistance training.

## 4. Materials and Methods

### 4.1. Sample Collection and Study Design

A cross-sectional study that included 282 individuals (195 males and 87 females) was performed on the general population of Mexico City. The eligibility criteria for study participation aligned with those used by the blood bank as a screening system for blood donation. These criteria required participants to meet the following conditions: being between 18 and 65 years of age, weighing more than 50 kg, and fasting for at least 8 h prior to participation. Exclusion criteria encompassed the following: a recent history of respiratory symptoms, diarrhea, or dental infections within the past 14 days; recent ingestion of drugs (including antibiotics, antidiarrheal medication, analgesics, etc.) in the five days leading up to the donation; recent endodontic procedures, acupuncture, piercings, or tattoos within the past 12 months; a surgical intervention within the last 6 months; vaccination within the last 30 days; consumption of alcoholic beverages within 72 h prior to donation; and insulin-dependent diabetes. The participants were subsequently asked about their alcohol consumption, smoking habits, family history of cancer, and whether they had been diagnosed with diabetes and/or hypertension (Table 1). The study included participants aged between 18 and 65 years, with an average age of 37.18 years and an average BMI of 27.75 kg/m^2^. Blood samples were obtained from the National Institute of Rehabilitation Luis Guillermo Ibarra Ibarra’s blood bank. The study received approval from Institutional Committees, and informed written consent was obtained from all participants. The participants in this study were identified as Mexican Mestizos, representing the majority of present-day Mexicans [27], based on anthropological criteria [35]. In this context, Mexican Mestizos are defined as individuals born in Mexico who do not associate with or identify as part of any indigenous group.

### 4.2. DNA Isolation and Genotyping

Genomic DNA was isolated from peripheral blood leukocytes by the DTAB-CTAB method [36]. The description of this method is provided so that other groups find it easier to reproduce the experiment: A blood sample of 5–10 mL was collected in a tube with EDTA (ethylenediamine tetra-acetic acid) as an anticoagulant, it was then centrifuged at 2000 rpm for 15 min to separate the leukocyte package. In one sterile 1.5 mL microcentrifugation tube (Eppendorf), 600 µL of 8% DTAB (dodecyl trimethylammonium bromide) (Sigma, NaCl 1.5 M, EDTA 50 mM, Tris 100 mM pH 8.7) (Saint Louis, MO, USA) was added and in another Eppendorf tube 100 µL of 5% CTAB (cetyltrimethylammonium bromide) (NaCl 0.4 M) was placed. Next, 150 µL of the leukocyte package (from the freshly centrifuged samples) was placed in the 1.5 mL Eppendorf tube containing 8% DTAB, the tube with the blood sample was centrifuged again at 2000 rpm for 15 min, and another 150 µL of the leukocyte package was obtained and deposited into the Eppendorf tube with DTAB from the previous step. The mixture was heated in a dry bath incubator at 65–68 °C for 5 min. After that, 550 µL of 100% Chloroform was added to the same Eppendorf tube and was manually agitated vigorously for 5 min; the mixture was then centrifuged at 13,000 rpm for 10 min. Following centrifugation, two phases were visible—a proteinaceous phase at the bottom of the tube and a transparent aqueous phase on the surface. The upper aqueous phase was poured into the tube containing CTAB, mixed, and 750 µL of sterile water was added. It was gently mixed until the phases were combined and then allowed to rest for 10 min at room temperature. Subsequently, it was centrifuged at 10,000 rpm for 5 min. The supernatant was discarded, retaining the precipitate, to which 200 µL of 1.2 M NaCl was added to resuspended it. Then, 850 µL of 100% cold ethanol was added, mixed, and allowed to precipitate in the cold for 10 min (at −20 °C). It was then centrifuged at 10,000 rpm for 5 min. The supernatant was discarded again and 500 µL of 70% ethanol was added. It was once more centrifuged at 10,000 rpm for 5 min, repeating the process of discarding the supernatant after. The remaining precipitate was dried in a vacuum dryer or at room temperature. Finally, the precipitate was resuspended in sterile water.

Agarose gels and a Nanodrop ND-100 were used to assess DNA quality before real-time PCR. A total of 20 ng of genomic DNA from peripheral blood was used for the PCR. Genotyping was performed with real-time PCR using TaqMan probes (hydrolysis probes) specific for the variants; *MSTN* (rs1805085, C_9480364_10, and rs1805086, C_282184_30), *BDKRB2* (rs1799722, C_1772941_10), *FST* (rs1423560, C_8884098_10), *ACTN3* (rs1815739, C_590093_1_), *ADRB2* (rs1042713, C_2084764_20, rs1042714, C_2084765_20), *GRB14* (rs8192673, C_1356985_10), *AGT* (rs699, C_1985481_20) and *EDN1* (rs5370, C_598677_1_) of Applied Biosystems, Foster City, CA, USA. PCR reactions were prepared using the standard conditions for 25 μL reactions, and the mixture was homogenized and placed in the dish; subsequently, we added 20 ng of DNA to each well. The reaction dish was sealed with adhesive film and gently centrifuged. The conditions for PCR were 95 °C, 10 min for the activation of the polymerase, 95 °C, 15 s for denaturation and an extension of 60 °C 1 min. The acquisition of data and allelic discrimination was carried out in LightCycler 480 II (Roche Diagnostics GmbH, Rotkreuz, Switzerland).

### 4.3. Statistical Analysis

Following the genotyping process, we conducted a thorough analysis of all the variants identified. Our primary objective was to determine whether these variants adhered to the Hardy–Weinberg Equilibrium (HWE) within our study population. The Hardy–Weinberg Equilibrium (HWE) is a statistical principle that describes the expected distribution of allele and genotype frequencies in a population under certain assumptions. These assumptions include random mating, no selection, no mutation and no migration. HWE is important for genetic association studies because it provides a baseline for comparing the observed allele and genotype frequencies in a study population to the expected frequencies under HWE. If the observed frequencies differ significantly from the expected frequencies, this suggests that one or more of the HWE assumptions has been violated. This could be due to a number of factors, such as population stratification, selection or genotyping errors. Therefore, we utilized a χ2 test, which is readily available online [37]. Once we had established the HWE status of our variants, we proceeded to compare the genotype and allele frequencies that we had obtained with those from twenty-six other populations. These populations were derived from the 1000 Genomes Project, a comprehensive resource that provides a detailed representation of human genetic variation.

For the statistical analysis, we employed SPSS software v. 18.0 (SPSS Inc., Chicago, IL, USA). This powerful tool allowed us to conduct a rigorous comparison of genotype and allele frequencies between our study population and the populations from the 1000 Genomes Project. We used the χ2 test in conjunction with Fisher’s exact test correction to compare these frequencies. This approach enabled us to identify any significant differences between populations. We set our *p*-value threshold at <0.05; any value below this threshold was considered indicative of statistically significant differences among populations.

### 4.4. Limitations of the Study

Although descriptive studies of allele frequencies in specific populations are a valuable tool for understanding the genetic diversity of human populations and identifying genetic variants that may be associated with disease or other traits, our study presented some limitations. These included a small sample size and a scarcity of information on individual characteristics, and we were unable to demonstrate why some of the variants were not in HWE; we limited our contribution to explaining how allele frequencies are important factors that may influence genetic case–control studies.

## Figures and Tables

**Table 1:** Comparison of demographic and clinical data of the study groups.

Variable	Female	Male	*p*-Value
Age	37.08 ± 11.75	37.23 ± 11.5	*p* = 0.96
Smoking			*p* = 0.017
(N)	60	125	
(Y)	18	79	
Alcoholism			*p* < 0.0001
(N)	59	88	
(Y)	19	116	
BMI	27.35 ± 4.37	27.91 ± 8.3	*p* = 0.25
Hypertension			*p* = 0.72
(N)	78	203	
(Y)	0	1	
Diabetes			*p* = 0.72
(N)	78	203	
(Y)	0	1	
Family History of Cancer			*p* = 0.05
(N)	51	157	
(Y)	27	47	

**Table 2:** Gene variants associated with exercise adaptation and complex metabolic diseases.

Gene Polymorphism (Variant Nucleotide)	Effect and/or Associations
*MSTN*	Muscle hypertrophy most associated with allele T [13,14]
rs1805085	
c.163C > T	
p.Ala55Thr	
*MSTN*	Muscle hypertrophy most associated with allele C [15]
rs1805086	
c.458T > C	
p.Lys153Arg	
*FST*	Association of the muscle size and the strength response to resistance training with allele T [16]
rs1423560	
c.-860G > T	
*BDKRB2*	Genotype TT implicated in endurance performance among habitual runners [17]
rs1799722	
c.-192C > T	
*ACTN3*	Runners’ performance associated with increases in muscle strength in response to resistance training CC [RR] genotype associated with increased strength TT [XX] genotype associated with increased endurance [18]
rs1815739	
c.1729C > T	
p.Arg577X	
*ADRB2*	G allele associates with elite athlete endurance performance in males A allele associates with increased BMI and decreased VO_2_ maximum [19,20,21]
rs1042713	
c.46G > A	
p.Gly16Arg	
*ADRB2*	Genotype CC contributes to metabolic syndrome and obesity susceptibility [22,23]
rs1042714	
c.79G > C	
p.Gln27Glu	
*GRB14*	Minor effect on carotid intima media thickness progression in the 3.8-year follow-up in Caucasians with T2DM Patients [24]
rs8192673	
c.1159 + 9C > T	
*AGT*	Polymorphism rs699 was associated with essential hypertension risk in Caucasians [25]
rs699	
c.803T > C	
p.Met268Thr	
*EDN1*	Homozygous TT genotype was associated with higher risk of hypertension [26]
rs5370	
c.594G > T	
p.Lys198Asn	

**Table 3:** Genotype and allele frequencies of the gene variants studied.

SNPS	Genotype Frequency			Allele Frequency		
		*n* = 282	%		*n* = 564	%
*MSTN*	CC	280	99.29	C	562	99.65
rs1805085	CT	2	0.71	T	2	0.35
*p* = 0.9523	TT	-	-			
						
*MSTN*	TT	270	95.74	T	551	97.70
rs1805086	TC	11	3.90	C	13	2.30
*p =* 0.0246	CC	1	0.36			
						
*FST*	GG	241	85.46	G	514	91.13
rs1423560	GT	32	11.35	T	50	8.87
*p* < 0.01	TT	9	3.19			
						
*BDKRB2*	CC	84	29.79	C	293	51.95
rs1799722	CT	125	44.32	T	271	48.05
*p =* 0.0597	TT	73	25.89			
						
*ACTN3*	CC	27	9.57	C	154	27.30
rs1815739	CT	100	35.47	T	410	72.70
*p* = 0.073	TT	155	54.96			
						
*ADRB2*	GG	80	28.37	G	302	53.55
rs1042713	AG	142	50.35	A	262	46.45
*p* = 0.8379	AA	60	21.28			
						
*ADRB2*	GG	7	2.48	G	65	11.52
rs1042714	GC	51	18.09	C	499	88.48
*p* = 0.0574	CC	224	79.43			
						
*GRB14*	CC	19	6.74	C	156	27.66
rs8192673	CT	118	41.84	T	408	7 2.34
*p* = 0.4436	TT	145	51.42			
						
*AGT*	GG	170	60.29	A	443	78.55
rs699	GA	103	36.52	G	121	21.45
*p* = 0.1596	AA	9	3.19			
						
*EDN1*	GG	218	77.30	G	497	88.12
rs5370	GT	61	21.63	T	67	11.88
*p* = 0.5774	TT	3	1.07			

The *p*-values under each gene indicate deviation from the expected values in the Chi-square test; *p* < 0.05 implies significant deviations.

## Data Availability

The data used to support the finding of this study are available from the corresponding author upon request.

## Supplementary Materials

The following supporting information can be downloaded at: https://www.mdpi.com/article/10.3390/muscles2040030/s1 Figure S1: Simulation of Case-Control Study Parameters [38].

## References

[1] Lozano R., Gomez-Dantes H., Garrido-Latorre F., Jimenez-Corona A., Campuzano-Rincon J.C., Franco-Marina F., Medina-Mora M.E., Borges G., Naghavi M., Wang H. (2013). Burden of disease, injuries, risk factors and challenges for the health system in Mexico. Salud Publica Mex..

[2] Costa A.M., Breitenfeld L., Silva A.J., Pereira A., Izquierdo M., Marques M.C. (2012). Genetic inheritance effects on endurance and muscle strength: An update. Sports Med..

[3] Lappalainen T., Scott A.J., Brandt M., Hall I.M. (2019). Genomic Analysis in the Age of Human Genome Sequencing. Cell.

[4] Peng C.K., Buldyrev S.V., Goldberger A.L., Havlin S., Sciortino F., Simons M., Stanley H.E. (1992). Long-range correlations in nucleotide sequences. Nature.

[5] Lohrer H.D., Tangen U. (2000). Investigations into the molecular effects of single nucleotide polymorphism. Pathobiol. J. Immunopathol. Mol. Cell. Biol..

[6] Ikeda Y. (1967). Genetic information theory—From the standpoint of molecular biology. Nihon Ishikai Zasshi. J. Jpn. Med. Assoc..

[7] Shastry B.S. (2009). SNPs: Impact on gene function and phenotype. Methods Mol. Biol..

[8] Appel M., Zentgraf K., Krüger K., Alack K. (2021). Effects of Genetic Variation on Endurance Performance, Muscle Strength, and Injury Susceptibility in Sports: A Systematic Review. Front. Physiol..

[9] Clos E., Pruna R., Lundblad M., Artells R., Esquirol Caussa J. (2019). ACTN3 single nucleotide polymorphism is associated with non-contact musculoskeletal soft-tissue injury incidence in elite professional football players. Knee Surg. Sports Traumatol. Arthrosc..

[10] Slager S.L., Schaid D.J. (2001). Case-control studies of genetic markers: Power and sample size approximations for Armitage’s test for trend. Hum. Hered..

[11] Goedecke J.H., Chorell E., van Jaarsveld P.J., Risérus U., Olsson T. (2020). Fatty acid metabolism and associations with insulin sensitivity differs between black and white South African women. J. Clin. Endocrinol. Metab..

[12] Varillas-Delgado D., Morencos E., Gutiérrez-Hellín J., Aguilar-Navarro M., Muñoz A., Mendoza Láiz N., Perucho T., Maestro A., Tellería-Orriols J.J. (2022). Genetic profiles to identify talents in elite endurance athletes and professional football players. PLoS ONE.

[13] Kollias H.D., McDermott J.C. (2008). Transforming growth factor-beta and myostatin signaling in skeletal muscle. J. Appl. Physiol..

[14] McPherron A.C., Lawler A.M., Lee S.J. (1997). Regulation of skeletal muscle mass in mice by a new TGF-beta superfamily member. Nature.

[15] Schuelke M., Wagner K.R., Stolz L.E., Hubner C., Riebel T., Komen W., Braun T., Tobin J.F., Lee S.J. (2004). Myostatin mutation associated with gross muscle hypertrophy in a child. New Engl. J. Med..

[16] Kostek M.A., Angelopoulos T.J., Clarkson P.M., Gordon P.M., Moyna N.M., Visich P.S., Zoeller R.F., Price T.B., Seip R.L., Thompson P.D. (2009). Myostatin and follistatin polymorphisms interact with muscle phenotypes and ethnicity. Med. Sci. Sports Exerc..

[17] Tsianos G.I., Evangelou E., Boot A., Zillikens M.C., van Meurs J.B., Uitterlinden A.G., Ioannidis J.P. (2010). Associations of polymorphisms of eight muscle- or metabolism-related genes with performance in Mount Olympus marathon runners. J. Appl. Physiol..

[18] Sarzynski M.A., Loos R.J., Lucia A., Perusse L., Roth S.M., Wolfarth B., Rankinen T., Bouchard C. (2016). Advances in Exercise, Fitness, and Performance Genomics in 2015. Med. Sci. Sports Exerc..

[19] Wagoner L.E., Craft L.L., Zengel P., McGuire N., Rathz D.A., Dorn G.W., Liggett S.B. (2002). Polymorphisms of the beta1-adrenergic receptor predict exercise capacity in heart failure. Am. Heart J..

[20] Wolfarth B., Rankinen T., Muhlbauer S., Scherr J., Boulay M.R., Perusse L., Rauramaa R., Bouchard C. (2007). Association between a beta2-adrenergic receptor polymorphism and elite endurance performance. Metab. Clin. Exp..

[21] Moore G.E., Shuldiner A.R., Zmuda J.M., Ferrell R.E., McCole S.D., Hagberg J.M. (2001). Obesity gene variant and elite endurance performance. Metab. Clin. Exp..

[22] Large V., Hellstrom L., Reynisdottir S., Lonnqvist F., Eriksson P., Lannfelt L., Arner P. (1997). Human beta-2 adrenoceptor gene polymorphisms are highly frequent in obesity and associate with altered adipocyte beta-2 adrenoceptor function. J. Clin. Investig..

[23] Dallongeville J., Helbecque N., Cottel D., Amouyel P., Meirhaeghe A. (2003). The Gly16→Arg16 and Gln27→Glu27 polymorphisms of beta2-adrenergic receptor are associated with metabolic syndrome in men. J. Clin. Endocrinol. Metab..

[24] Pleskovic A., Santl Letonja M., Cokan Vujkovac A., Starcevic J.N., Petrovic D. (2016). Polymorphisms of the PPAR-gamma (rs1801282) and Its Coactivator (rs8192673) Have a Minor Effect on Markers of Carotid Atherosclerosis in Patients with Type 2 Diabetes Mellitus. PPAR Res..

[25] Bonfim-Silva R., Guimaraes L.O., Souza Santos J., Pereira J.F., Leal Barbosa A.A., Souza Rios D.L. (2016). Case-control association study of polymorphisms in the angiotensinogen and angiotensin-converting enzyme genes and coronary artery disease and systemic artery hypertension in African-Brazilians and Caucasian-Brazilians. J. Genet..

[26] Rankinen T., Church T., Rice T., Markward N., Leon A.S., Rao D.C., Skinner J.S., Blair S.N., Bouchard C. (2007). Effect of endothelin 1 genotype on blood pressure is dependent on physical activity or fitness levels. Hypertension.

[27] Moreno-Estrada A., Gignoux C.R., Fernandez-Lopez J.C., Zakharia F., Sikora M., Contreras A.V., Acuna-Alonzo V., Sandoval K., Eng C., Romero-Hidalgo S. (2014). Human genetics. The genetics of Mexico recapitulates Native American substructure and affects biomedical traits. Science.

[28] Fairley S., Lowy-Gallego E., Perry E., Flicek P. (2020). The International Genome Sample Resource (IGSR) collection of open human genomic variation resources. Nucleic Acids Res..

[29] Rubi-Castellanos R., Martinez-Cortes G., Munoz-Valle J.F., Gonzalez-Martin A., Cerda-Flores R.M., Anaya-Palafox M., Rangel-Villalobos H. (2009). Pre-Hispanic Mesoamerican demography approximates the present-day ancestry of Mestizos throughout the territory of Mexico. Am. J. Phys. Anthropol..

[30] Hong E.P., Park J.W. (2012). Sample size and statistical power calculation in genetic association studies. Genom. Inf..

[31] Li J.J., Abecasis G. GAS Power Calculator. https://csg.sph.umich.edu/abecasis/gas_power_calculator/index.html.

[32] Royo J.L. (2021). Hardy Weinberg Equilibrium Disturbances in Case-Control Studies Lead to Non-Conclusive Results. Cell J..

[33] Jones M.R., Wilson S.G., Mullin B.H., Mead R., Watts G.F., Stuckey B.G. (2007). Polymorphism of the follistatin gene in polycystic ovary syndrome. Mol. Hum. Reprod..

[34] dbSNP Short Genetic Variations. https://www.ncbi.nlm.nih.gov/projects/SNP/snp_ss.cgi?subsnp_id=ss2229352.

[35] Curiel-Cervantes V., Solís-Sáinz J.C., Costa-Urrutia P., Aguilar-Galarza A., Flores-Viveros K.L., García-Gasca T.J., Anaya-Loyola M.A. (2020). The myostatin rs1805086 variant is associated with obesity in Mexican adults, independently of metabolic risk factors. Biomarkers.

[36] Gustincich S., Manfioletti G., Del Sal G., Schneider C., Carninci P. (1991). A fast method for high-quality genomic DNA extraction from whole human blood. Biotechniques.

[37] Pearl S., Dorothy A. Hardy-Weinberg Equilibrium Calculator for 2 Alleles. https://www.had2know.org/academics/hardy-weinberg-equilibrium-calculator-2-alleles.html.

[38] Campos-Nonato I., Oviedo-Solís C., Vargas-Meza J., Ramírez-Villalobos D., Medina-García C., Gómez-Álvarez E., Hernández-Barrera L., Barquera S. (2023). Prevalencia, tratamiento y control de la hipertensión arterial en adultos mexicanos: Resultados de la Ensanut 2022. Salud Pública México.

