# Supplementary material for: Prevalence of Ten Gene Variants Involved in Muscular Phenotypes in a Mexican Mestizo Population
Source: Muscles. 2023 Dec 8;2(4):389–99. doi: 10.3390/muscles2040030 (PMC12225391; doi:10.3390/muscles2040030)
Supplement: Supplementary file 1 [file muscles-02-00030-s001.zip › muscles-2629584-supplementary.pdf]

SUPPLEMENTARY MATERIAL. FIGURE S1 SIMULATION OF CASE-CONTROL STUDY PARAMETERS

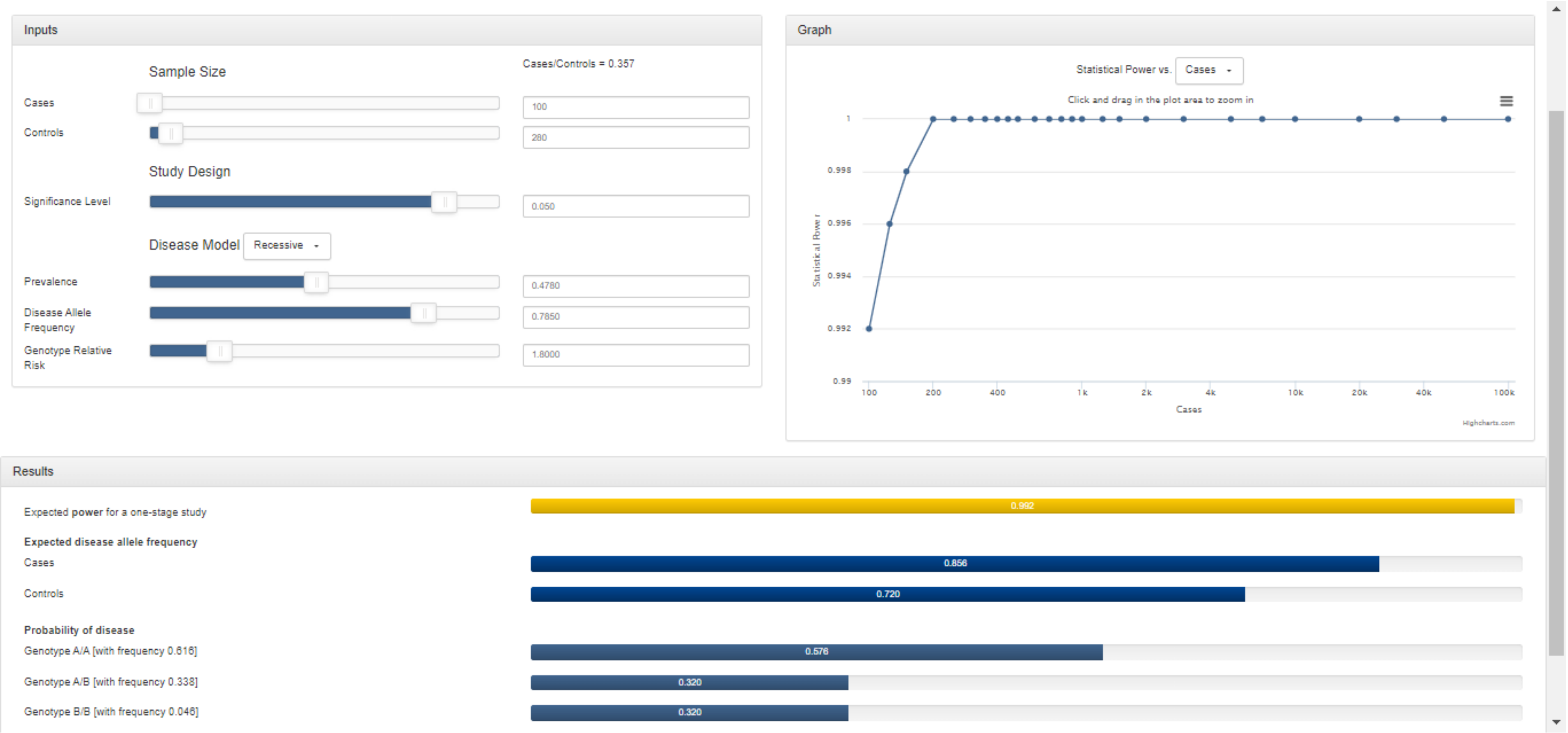

A simulation was performed with the prevalence of hypertension in Mexicans of 47.8%, according to what was published by Campos-Nonato et al. Taking the odds ratio published by Bonfim-Silva (OR=1.8), we subsequently introduced data from the present article, such as the frequency of the risk allele.

1. Bonfim-Silva, R.; Guimaraes, L.O.; Souza Santos, J.; Pereira, J.F.; Leal Barbosa, A.A.; Souza Rios, D.L. Case-control association study of polymorphisms in the angiotensinogen and angiotensin-converting enzyme genes and coronary artery disease and systemic artery hypertension in African-Brazilians and Caucasian-Brazilians. Journal of genetics 2016, 95, 63-69.
2. Campos-Nonato I, Oviedo-Solís C, Vargas-Meza J, Ramírez-Villalobos D, Medina-García C, Gómez-Álvarez E, Hernández-Barrera L, Barquera S. Prevalencia, tratamiento y control de la hipertensión arterial en adultos mexicanos: resultados de la Ensanut 2022. Salud Publica Mex [Internet]. 14 de junio de 2023 [citado 7 de noviembre de 2023];65:s169-s180. Disponible en: https://saludpublica.mx/index.php/spm/article/view/147
